# Multifunctionalized Reduced Graphene Oxide Biosensors for Simultaneous Monitoring of Structural Changes in Amyloid-β 40

**DOI:** 10.3390/s18061738

**Published:** 2018-05-28

**Authors:** Dahye Jeong, Jinsik Kim, Myung-Sic Chae, Wonseok Lee, Seung-Hoon Yang, YoungSoo Kim, Seung Min Kim, Jin San Lee, Jeong Hoon Lee, Jungkyu Choi, Dae Sung Yoon, Kyo Seon Hwang

**Affiliations:** 1 Department of Clinical Pharmacology and Therapeutics, College of Medicine, Kyung Hee University, Seoul 02447, Korea; dahye303@gmail.com (D.J.); bechu88@gmail.com (M.-S.C.); 2 Department of Chemical and Biological Engineering, Korea University, Seoul 02841, Korea; jungkyu_choi@korea.ac.kr; 3 Department of Biomedical Engineering, Dongguk University, Seoul 04620, Korea; lookup2@dongguk.edu; 4 Department of Biomedical Engineering, Yonsei University, Wonju 26493, Korea; lws729@gmail.com; 5 Systems Biotechnology Research Center, Korea Institute of Science and Technology (KIST), Gangnueung 25451, Korea; shyang@kist.re.kr; 6 Department of Pharmacy & Intergrated Science and Engineering Division, Yonsei University, Incheon 21983, Korea; y.kim@yonsei.ac.kr; 7 Center for Institute of Advanced Composite Materials, Korea Institute of Science and Technology (KIST), Jeonbuk 55324, Korea; seungmin.kim@kist.re.kr; 8 Department of Neurology, Kyung Hee University Hospital, Seoul 02447, Korea; xpist@naver.com; 9 Department of Electrical Engineering, Kwangwoon University, Seoul 01897, Korea; jhlee@kw.ac.kr; 10 Department of Biomedical Engineering, Korea University, Seoul 02841, Korea

**Keywords:** reduced graphene oxide(rGO), Alzheimer’s disease, amyloid beta, biosensor

## Abstract

Determination of the conformation (monomer, oligomer, or fibril) of amyloid peptide aggregates in the human brain is essential for the diagnosis and treatment of Alzheimer’s disease (AD). Accordingly, systematic investigation of amyloid conformation using analytical tools is essential for precisely quantifying the relative amounts of the three conformations of amyloid peptide. Here, we developed a reduced graphene oxide (rGO) based multiplexing biosensor that could be used to monitor the relative amounts of the three conformations of various amyloid-β 40 (Aβ40) fluids. The electrical rGO biosensor was composed of a multichannel sensor array capable of individual detection of monomers, oligomers, and fibrils in a single amyloid fluid sample. From the performance test of each sensor, we showed that this method had good analytical sensitivity (1 pg/mL) and a fairly wide dynamic range (1 pg/mL to 10 ng/mL) for each conformation of Aβ40. To verify whether the rGO biosensor could be used to evaluate the relative amounts of the three conformations, various amyloid solutions (monomeric Aβ40, aggregated Aβ40, and disaggregated Aβ40 solutions) were employed. Notably, different trends in the relative amounts of the three conformations were observed in each amyloid solution, indicating that this information could serve as an important parameter in the clinical setting. Accordingly, our analytical tool could precisely detect the relative amounts of the three conformations of Aβ40 and may have potential applications as a diagnostic system for AD.

## 1. Introduction

Amyloid-β (Aβ) peptides, including Aβ40 and Aβ42, are crucial hallmarks of Alzheimer’s disease (AD), and excessive production of Aβ and its deposition at the brain surface cause the various symptoms of AD [1,2]. The species and relative ratios of Aβ are indicators of the progression and stage of AD or the dependence of the condition on family history [3,4], respectively. Two major isoforms of Aβ, i.e., Aβ40 and Aβ42, are generally considered key peptides in the diagnosis of AD [5,6,7,8]. Although the concentrations of both Aβ40 and Aβ42 are related to the progression of AD [9,10], accurate measurement of Aβ40 is particularly important. Moreover, even if total Aβ levels are increased, the Aβ42 level in the Cerebrospinal fluid (CSF) can decrease unexpectedly due to deposition of aggregated Aβ42 in the brain [10,11]. Patients with AD also have approximately 10-fold more Aβ40 than Aβ42 in the CSF [4], and a considerable amount of Aβ is released through the blood brain barrier, allowing it to be distributed throughout the body. Accordingly, the detection of Aβ40 levels may be an excellent strategy for blood-based examination of nonfamilial AD because of the higher concentration of Aβ40 in serum [8]. Accurate detection of Aβ40 is also necessary to correctly diagnose familial AD. Indeed, the total amounts of both Aβ42 and Aβ40 are lower in familial AD than in nonfamilial AD [4,10]. Accordingly, the concentration ratio of Aβ42/Aβ40 is relevant to the diagnosis of AD, and accurate detection of Aβ40 is important for estimation of this ratio [12,13].

Many research groups have investigated how the major conformation (monomer, oligomer, or fibril) of Aβ is strongly related to AD progression and toxicity [14,15]. Moreover, the Aβ oligomer contributes substantially to neurotoxicity [16] and leads to the death of neural cells. Accordingly, manipulation of Aβ aggregation through chemical injection may be an effective treatment for AD [17]. Kim and co-workers have reported that amyloid plaques are rapidly disaggregated following treatment with 4-(2-hydroxyethyl)-1-piperazinepropanesulphonic acid (EPPS), resulting in alleviation of symptoms in AD model mice [18]. In addition to these effects, EPPS also disaggregates neuro-toxic fibrils and oligomers into nontoxic monomers, further facilitating recovery in AD model mice. Accurate monitoring of Aβ aggregation and disaggregation, as well as evaluation of the amounts of Aβ40 with particular conformations present in body fluid, is essential in the diagnosis and treatment of AD.

In our recent research, Kim and co-workers reported possibility of the measuring Aβ42 level in the blood of an AD (APP/PS1) mouse model with reduced graphene oxide (rGO) biosensor [19,20]. Accordingly, although there were previously reported novel rGO biosensor to detect Aβ42 [19,20], the development of a smart biosensor capable of simultaneous detection of all possible conformations of Aβ is necessary to obtain important information regarding the relative amounts of these specific peptides (monomers, oligomers, and fibrils).

**Figure 1:**
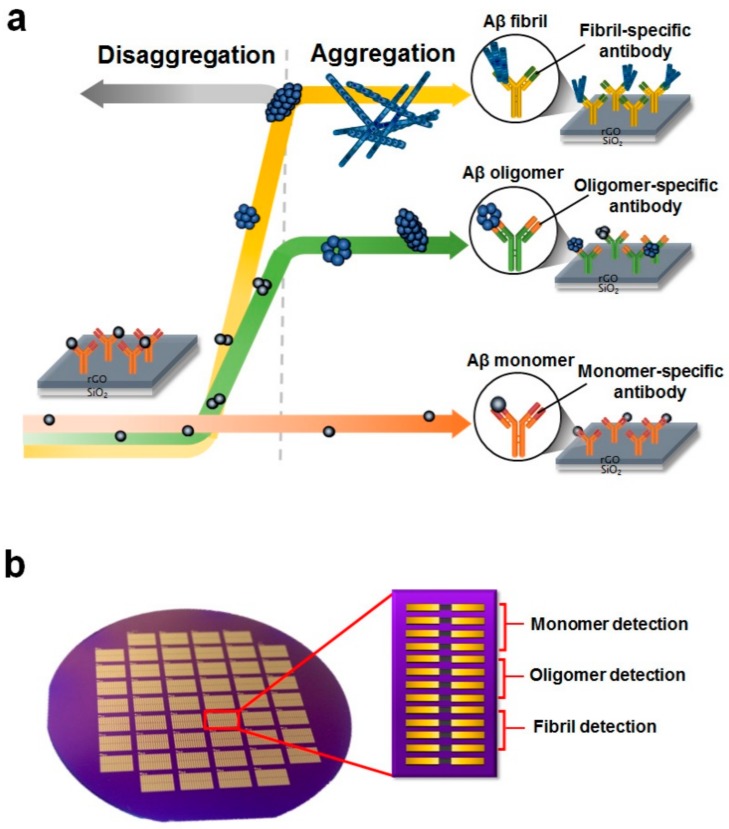
Device structure of a single reduced graphene oxide (rGO) sensor and its detection scheme. (**a**) Illustration of Aβ aggregation and its detection principle. During incubation, the Aβ40 monomers aggregated, forming oligomers and fibrils. Relative amounts of the three conformations of Aβ40 were examined by antigen-antibody interactions using corresponding antibodies for each conformation; (**b**) An optical image of the rGO-based biosensor. Wafer-scale rGO patterned sensors were developed by conventional microelectromechanical (MEMS) system techniques. In one 4-inch wafer, there were 50 devices, and each device had 13 rGO sensor units. The first four units were for monomer detection, the second four units were for oligomer detection, and the third set of four units was for fibril detection. The remaining one unit was used as the reference sensor.

In this report, we developed a rGO biosensor that could be used to monitor the relative amounts of the three conformations in various amyloid fluids (Figure 1a). The rGO biosensor was composed of a multichannel sensor array capable of individual detection of monomers, oligomers, and fibrils in a single amyloid fluid (Figure 1b). The corresponding antibodies for detecting monomers, oligomers, and fibrils were immobilized on each rGO surface in the sensor. The performance of each rGO sensor element was tested by measuring the conductance change when the Aβ sample added. To verify whether the rGO biosensor could be used to evaluate the relative amounts of the three conformations of Aβ40, various amyloid solutions (monomeric Aβ40, aggregated Aβ40, and disaggregated Aβ40 solutions) were employed. Our findings confirmed that the relative amounts of the three conformations varied significantly with respect to the different amyloid solutions. Information regarding the relative amounts of the three conformations of Aβ40 could provide us with important insights into the distributions of these specific Aβ40 conformations in the body fluids of patients. Accordingly, our analytical tool may have potential applications as a diagnostic system for AD.

## 2. Materials and Methods

### 2.1. Materials

Recombinant amyloid beta peptide 1–40 (Aβ40; amino acid sequence: NH_2_-DAEFRHDSGYEVHHQKLVFFAEDVGSNKGAIIGLMVGGVV-COOH, human; Tocris Bioscience, Bristol, UK) was utilized to fabricate Aβ40 solutions with different combinations of the three conformations (monomers, oligomers, and fibrils). We employed three different types of antibodies as receptors for capturing monomers, oligomers, and fibrils of Aβ40, including monoclonal 6E10 antibodies (Covance, Princeton, NJ, USA) specific for Aβ sequence 1–16, polyclonal A11 antibodies (200-401-E88l Rockland, ME, USA) for Aβ40 oligomers, and polyclonal OC antibodies (200-401-E87, Limerick, PA, USA) for Aβ40 fibrils. Each type of antibody was immobilized on the individual sensor unit in the rGO sensor to detect the corresponding conformation of Aβ40. All antibodies were immobilized at the carboxyl reaction sites of rGO by coupling with N-(3-dimethylaminopropyl)-N′-ethylcarbodiimide hydrochloride (EDC) and N-hydroxysuccinimide (NHS).

Various chemicals were employed to fabricate the Aβ40 solutions with different combinations of monomers, oligomers, and fibrils. For example, DMSO (Sigma-Aldrich, St. Louis, MO, USA) and PBS (pH 7.8, 1×; Corning, NY, USA) were used as first dilution media for preparing aggregated forms of Aβ40. The disaggregation of Aβ oligomers and fibrils in the solution was accomplished by the addition of EPPS (pH 7.3–8.7; Sigma-Aldrich, St. Louis, MO, USA), which converts Aβ aggregates into monomers.

To obtain rGO films, we used an aqueous GO solution (SKU-HCGO-W-175; Graphene Supermarket, New York, NY, USA). The solution was diluted with ultrapure Milli-Q water, and its concentration was adjusted to 3.25 mg/mL prior to deposition. A motorized stage (AL1-1515-3S; Micro Motion Technology, Seoul, Korea) was used to control the movement of the deposition plate. The GO film on the substrate was fabricated by injecting the GO solution and moving the plate with a constant speed of 20 mm/s. The GO film was chemically reduced with hydroiodic acid (HI) vapor at 80 °C for 3 h, resulting in the transformation into rGO.

### 2.2. Methods

#### 2.2.1. Preparation of Aβ40 Aggregates with Various Conformations

**Figure 2:**
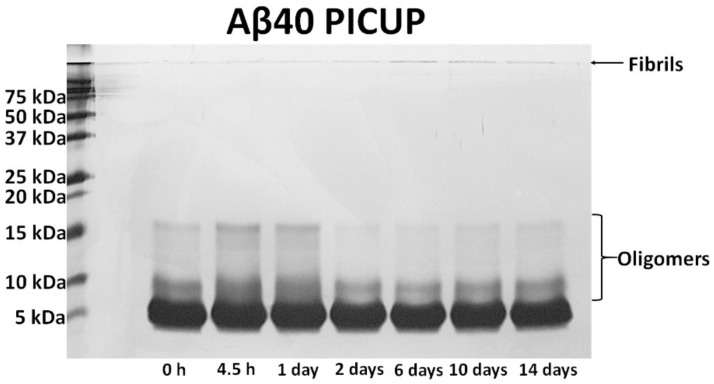
Verification of time-dependent Aβ aggregation using the photo-induced crosslinking of unmodified protein (PICUP) method. To visualize Aβ monomers, oligomers, and fibrils by gel electrophoresis, we used SDS-PAGE and PICUP chemistry. The Aβ40 peptide solutions were incubated at 37 °C for 4.5 h, 1 day, 2 days, 6 days, 10 days, or 14 days. The Aβ_40_ samples were then quickly irradiated twice (each for 1 s) for crosslinking the peptides with Ru(Bpy)(Cl_2_) and ammonium persulfate. The crosslinked Aβ samples were analyzed on 15% Tris-glycine gels. After electrophoresis, highly sensitive silver staining was performed for visualizing Aβ40 bands in the gel.

Monomeric Aβ40 peptide was firstly prepared by dis-solving lyophilized Aβ peptide into DMSO (≥99.5% purity) to a concentration of approximately 1 mg/mL Aβ40. The dissolved Aβ monomer was serially diluted with PBS 1× in order to obtain solutions with different Aβ40 concentrations ranging from 100 fg/mL to 10 ng/mL. Oligomers and fibrils were fabricated by incubating the monomeric Aβ40 solution at 37 °C for various times (4.5 h, 1 day, 2 days, 6 days, and 10 days). During the incubation, the solutions were continuously stirred and periodically vortexed every 24 h. The resulting samples were kept on ice and completely consumed within 30 min for various experiments with regard to the rGO biosensors. For structural analysis of aggregated Aβ40, each incubated Aβ40 solution was examined by TEM and photo-induced crosslinking of unmodified proteins (PICUP) (Figure 2).

#### 2.2.2. Disaggregation of Aβ40 Peptides

The Aβ40 solution incubated for 10 days was employed to observe the disaggregation behaviors of the Aβ40 aggregates by addition of EPPS. The Aβ40 concentration of the solution was adjusted to 10 ng/mL by dilution with PBS 1x. The solution contained large amounts of fibrils and few monomers or oligomers. EPPS was dissolved in PBS 1×, and its concentration was adjusted to 10 mM. The aggregated Aβ40 solution was blended with the EPPS solution to a volume ratio of 4:1. The mixed solution of EPPS and aggregated Aβ40 was incubated at room temperature (20 to 25 °C) for 24 h [18]. Structural changes in the resulting Aβ40 were quantitatively measured by atomic force microscopy (AFM) (Multimode V, Veeco Instruments, Inc, Plainview, NY, USA) and analyzed using image processing software (NanoScope Analysis 1.50).

### 2.3. Antibody Immobilization and Reaction with Aβ40 Peptides

Antibodies for capturing monomers (6E10), oligomers (A11), and fibrils (OC) were diluted with PBS 1×, and the antibody concentration of each solution was adjusted to 10 μg/mL. After dilution, each antibody was immobilized onto individual rGO sensor units in the device with 2 mM EDC and 8 mM NHS for 2 h. To remove the residual anti-bodies, all rGO sensors were washed with 2 mL of PBS 1× and 3 mL deionized (DI) water and subsequently dried with pure nitrogen gas. These antibodies were chemically immobilized on the RGO surfaces to recognize the corresponding conformation of Aβ40. The amine group (-NH_2_) of the antibodies were covalently functionalized to oxygenated groups (e.g., carboxyl group) via the *N*-(3-dimethylaminopropyl)-*N*′-ethylcarbodiimide hydrochloride (EDC) and *N*-hydroxysuccinimide (NHS) coupling reaction. The confirmation of antibody immobilization has been investigated in previous studies according to changes in the electrical characteristic and surface morphologies before and after these interactions [19,20]. This individual immobilization enabled the fabrication of rGO sensors capable of simultaneous detection of all conformations of Aβ40. These antibody molecules were generally stored and reacted with different type of Aβ analytes in aqueous environment based on PBS 1× buffer solution (pH 7.4). In order to acquire electrical signals before and after specific reactions, the antibody that was immobilized on RGO surfaces was exposed to the ambient temperature and pressure during short period of measurement time, within 5 min. Several literature have already claimed the long-term stability and reactivity of the air-dried antibody maintained when exposed to ambient conditions [21,22], and our previous studies also supported this [19,20]. Thus, the experimental condition in this study was deemed sufficient to in vitro assay for protein recognition.

### 2.4. Fabrication of rGO Sensor

#### 2.4.1. Formation of rGO Films

The rGO thin films were formed on SiO_2_/Si substrates by GO deposition and subsequent reduction. A schematic of the overall fabrication is shown in Figure S1. As shown in our previous studies [19,20], same purchased GO solution which is made by exfoliation of GO flakes by Hummers method [23,24] and dispersed in ultrapure Milli-Q water [25] was utilized. Using the meniscus-dragging deposition (MDD) technique [26], the GO films were deposited on the 4-inch SiO_2_ (300 nm)/Si substrates (Figure S1a). To deposit the GO thin films, a glass plate (127 × 127 mm^2^) cleaned with a piranha solution (H_2_SO_4_/H_2_O_2_ = 3:1) was used. One hundred microliters of the GO solution was dropped between the SiO_2_/Si substrate and the glass plate, which was positioned at a contact angle of 30° with respect to the substrate. A motorized stage was used to spread the GO solution on the substrate at a constant speed of 20 mm/s. The deposition process was repeated 20 times to obtain the desired thickness. After the deposition, chemical reduction of the GO thin films was performed with hydroiodic acid (HI) vapor at 80 °C for 3 h. The final thick-ness of the rGO thin film was approximately 1 μm.

#### 2.4.2. Fabrication of rGO Biosensor

For specific detection of conformational changes in Aβ40, our sensor system was based on electrical measurement in which the resistance of the rGO thin film was monitored when Aβ40 was added. Here, we employed rGO films as sensing interfaces of transformation from biomolecular interactions (e.g., Aβ40-antibody binding) to electrical signals. As shown in Figure S1b, the rGO sensors were fabricated according to the standard microelectromechanical system (MEMS) procedure developed and validated in our laboratory [19,20]. A photoresist (PR) film (GXR601, AZ Electronic Materials, Somerville, NJ, USA) was spin coated on the rGO film at 3000 rpm for 30 s. The PR pattern indicative of the sensor array on the substrate was developed through a photolithography process. Subsequently, the rGO sensor array was formed by reactive ion etching (VISION 320 RIE, Advanced Vacuum & STS, Järngatan, Sweden) performed at 300 W for 30 s in an O_2_ atmosphere. The gold electrodes as contact pads were formed through photolithography, Au deposition, and the subsequent lift-off process. Specifically, a gold layer (~200 nm) with a Cr adhesive layer (50 nm) was deposited on the PR-patterned rGO/SiO_2_ substrate using an electron-beam evaporator. The remaining PR was removed with acetone, and the substrate was continually washed with methanol, isopropyl alcohol, and deionized (DI) water. Each patterned rGO film had dimensions of 100 × 50 µm^2^ for a single sensor unit on which antibodies for capturing Aβ40 were immobilized after oxygen plasma treatment. A single rGO device had 13 sensor units, and 50 devices were present in a 4-inch wafer.

#### 2.4.3. Performance Test of the rGO Sensors with Various Aβ40 Solutions

Detailed information regarding the performance test was described previously [19]. Briefly, 80 µL of each Aβ40 solution was dispended onto the rGO sensor and then incubated at 25 °C for 20 min for the Aβ40-antibody interaction. After the bioassay, the rGO sensor was washed with 2 mL of PBS 1× and 3 mL of DI water. For electrical measurement, all rGO sensors were dried with pure nitrogen gas. The resistance of the rGO sensor was measured using a semiconductor analyzer (B1500A, Agilent, Santa Clara, CA, USA) combined with a probe station (AP-8000, Unitek Corp., Seoul, Korea).

## 3. Results and Discussion

### 3.1. General Measuring Principle of the rGO Sensor

For the exact detection of Aβ40-antibody interactions, our sensor system was based on electrical measurement in which the resistance of the rGO thin film was monitored. When the Aβ40 solution was added, changes in the resistance of each rGO sensor unit due to biomolecular inter-actions were measured using a semiconductor analyzer combined with a probe station. In this study, adopting the concept of relative resistance changes helped us to easily handle our measured results [27,28]. The relative resistance change (ΔR_1_) caused by antibody immobilization is defined as (R_ab_ − R)/R, where R is the initial resistance of the bare rGO unit, and R_ab_ represents the measured resistance of the unit after antibody immobilization. In an analogous way, the relative resistance change (ΔR_2_) caused by the Aβ40-antibody interaction could also be defined as (R_rxn_ − R_ab_)/R_ab_, where R_rxn_ is the measured resistance of the unit after the Aβ40-antibody interaction.

After antibody immobilization, the ΔR_1_ values of the rGO units due to 6E10, A11, and OC antibodies were evaluated (Figure S2). The rGO sensor units immobilized with 6E10, A11, and OC antibodies showed significant increases in resistance, exhibiting ΔR_1_ values of 17.24%, 13.21%, and 13.62%, respectively. The ΔR_1_ values of all the units were narrowly distributed with high uniformity, implying that reliable biorecognition of Aβ40 was able to be accomplished using our devices.

### 3.2. Aβ Aggregation of Aβ40 Peptides with Respect to Incubation Time

We fabricated various Aβ40 solutions with different incubation times and examined the conformations of the Aβ40 by transmission electron microscopy (TEM). The Aβ40 monomer was diluted with 1% (*v/v*) dimethyl sulfoxide (DMSO) in 1× phosphate-buffered saline (PBS) to a concentration of 10 μg/mL, 1% (*v/v*) DMSO in PBS has been shown to be the most suitable buffer for the formation of oligomers and fibrils, and higher concentrations of DMSO hinder the aggregation of Aβ [29]. To obtain Aβ40 aggregates with different conformations, the Aβ40 solutions were incubated at 37 °C for 0 (no incubation), 6, and 10 days. We hypothesized that the majority of Aβ40 would exist as monomers without incubation and that the portions of oligomers and fibrils would gradually increase as the incubation time was increased.

**Figure 3:**
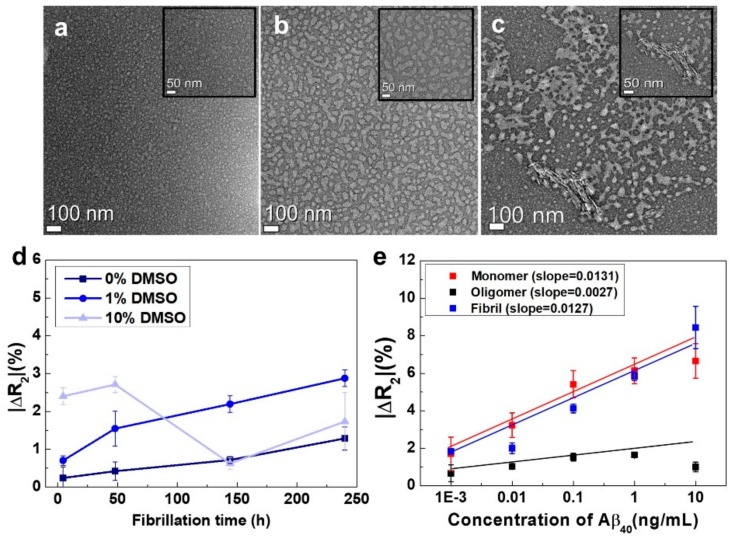
(**a**) Structural characterization of Aβ40 monomers; (**b**) oligomers; and (**c**) fibrils by conventional TEM. TEM images were taken from Aβ40 samples with (**a**) no incubation; (**b**) incubation at 37 °C for 6 days; and (**c**) incubation at 37 °C for 10 days; (**d**) The aggregation characteristics of Aβ40 solutions with different DMSO concentrations. To estimate the extent of Aβ40 aggregation, we employed rGO sensors with OC antibodies, which specifically interacted with the fibrils. The ΔR_2_ values of the rGO sensors were measured when Aβ40 solutions with different incubation times were added at each DMSO concentration (*n* = 12); (**e**) Performance test of the rGO sensors with respect to the concentration of each conformation of Aβ40 (*n* = 7).

To test this hypothesis, we conducted TEM analysis using the Aβ40 solutions (Figure 3a–c). For the Aβ40 solution without incubation, a number of small features were found scattered in the image, measuring ~10 nm in width. Thus, the majority of Aβ40 remained in the monomeric form, and there were no fibrils at this time point. The TEM image of the Aβ40 incubated for 6 h showed the presence of elongated fibrous shapes measuring 50–100 nm in width. Small features (~10 nm) corresponding to the monomeric form of Aβ40 were rarely observed in the image. Thus, the majority of Aβ40 formed oligomers, and very few monomers and fibrils coexisted in the solution. In contrast, the Aβ40 sample incubated for 10 h showed larger elongated features with a high aspect ratio, indicating that the Aβ40 aggregates formed fibrils in larger amounts. Moreover, the particular features (about 10 nm and 50–80 nm in width) corresponding to monomers and oligomers were still observed in the image, regardless of the long incubation time. In addition, Thioflavin T (ThT) fluorescent assay was performed to confirm the formation of Aβ40 fibrils. ThT intensities of all the samples were measured using multilabel plate readers (PerkinElmer, Waltham, MA, USA) with a 430 nm excitation filter and a 480 nm emission filter. As shown in Figure S3, The ThT intensities of Aβ40 before and after incubation were 7042 ± 142 and 7313 ± 173 in arb. units, respectively. The increased ThT intensity of the Aβ40 sample with incubation strongly supports the formation of β-sheets-rich aggregates (i.e., fibrils) during incubation. The AFM analysis was also carried out to confirm whether the Aβ40 formed actual amyloid fibrils after incubation. Based on the AFM image, it was observed that the sample (10 ng/mL Aβ40) with incubation had a large amount of short Aβ40 fibrils with approximately several hundred nanometers or less. According to these results, it was obviously confirmed that Aβ40 fibrils were formed after incubation using conventional methods.

Verifying whether the aggregation behavior of Aβ40 could also be monitored using our rGO sensors was a challenging task necessary to prove the reliability of our device. rGO sensors immobilized with OC antibodies for capturing the fibrils were employed to monitor the extent of Aβ40 fibrillation in each solution. We measured the ΔR_2_ values of the rGO sensors when Aβ40 samples with different DMSO concentrations (0%, 1%, and 10%) and different incubation times were added. Figure 3d depicts the relative resistance changes of the rGO sensors when the Aβ40 samples were added. At 1% DMSO, the ΔR_2_ of the rGO sensor rapidly increased as the incubation time increased, indicating that Aβ40 fibrillation actively occurred in 1% DMSO buffer. This trend was consistent with the results of TEM analysis obtained from the same solutions (Figure 3a–c). Accordingly, the aggregation behavior of Aβ40 could be easily monitored using our devices without complicated and time-consuming analysis, such as that required for TEM. In the case of 0% DMSO, the ΔR_2_ of the rGO sensor moderately increased with incubation time, exhibiting a slight slope. This suppression of Aβ40 fibrillation in the absence of DMSO could be attributed to the observation that DMSO causes homogeneous dispersion of Aβ40 peptides due to their relatively hydrophobic nature [30,31]. However, the addition of high concentrations of DMSO may result in inconsistent results with regard to Aβ40 fibrillation. Indeed, for Aβ40 samples treated with 10% DMSO, there was no consistent trend in ΔR_2_, implying that there was an optimal concentration of DMSO for Aβ40 fibrillation. Thus, subsequent experiments evaluating incubation time for Aβ40 fibrillation were conducted using 1% DMSO.

### 3.3. Basic Characterization of the rGO Sensors, Including Their Sensitivity and Selectivity with Respect to Each Conformation of Aβ40 Peptide

For the sensitivity test, we needed to prepare model solutions consisting of pure monomers, oligomers, and fibrils. However, it is extremely difficult to obtain such pure solutions. Instead, we prepared three test solutions, each of which contained a particular conformation of the Aβ40 peptide in a very large amount. The test solution for the monomers was prepared by dissolving Aβ40 powder in PBS. We had already confirmed that this solution contained primarily Aβ40 monomers (Figure 3a). The test solution for the fibrils was made through microdialysis [27] of the Aβ40 solution after incubation for 10 days. The solution after microdialysis contained fibrils in large amounts because the majority of monomers and oligomers diffused outward during the process (the fibrils without EPPS will be discussed in Section 3.4). We employed the solution after 6 days of incubation as the test solution for the oligomers. We also confirmed that the solution with 6 days of incubation contained oligomers in the majority (Figure 3b). Figure 3e shows quantitative analyses of the three Aβ40 solutions with different concentrations using the rGO sensors. From the results, we found that the ΔR_2_ value of the sensor was proportional to the concentration (1 pg/mL to 10 ng/mL) of each conformation, both for monomers and fibrils. The ΔR_2_ varied from 1.8% to 8.4%, and the sensor for the oligomers also showed a linear relationship with respect to fibril concentration (1 pg/mL to 1 ng/mL). For the 10 ng/mL oligomer solution, however, the ΔR_2_ decreased slightly. The reason for this observation is still unclear, and additional studies are needed. Based on these results, we concluded that our sensors had excellent analytical sensitivity (~1 pg/mL) and a fairly good dynamic range (1 pg/mL to 1–10 ng/mL) as shown in Table S1. This suggested that relative amounts of the three conformations of Aβ40 could be successfully analyzed using our sensors.

**Figure 4:**
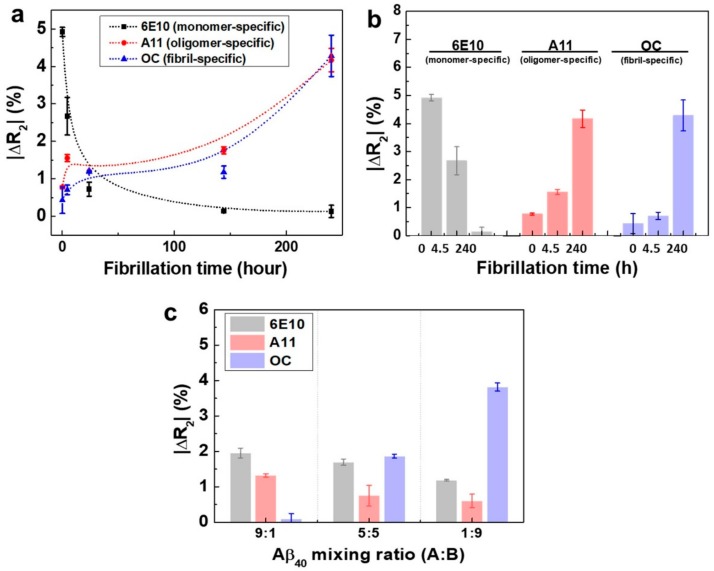
(**a**) Monitoring of the aggregation behavior of the Aβ40 solution with respect to the incubation time. All the incubation experiments were conducted using the 1 ng/mL Aβ40 monomer solution (*n* = 12); (**b**) The ΔR_2_ values of the rGO sensors at three different incubation times (no incubation, 4.5 h, and 10 days) (*n* = 12); (**c**) Selectivity test of rGO sensors by mixing both the monomer-rich (A) and fibril-rich (B) solutions.

The ability to evaluate the relative amounts of the three conformations (monomers, oligomers, and fibrils) of Aβ in a particular solution using a single device is essential and would greatly simplify the cumbersome processes required to perform individual detection of each Aβ40 conformation, thereby having benefits for clinical applications. Accordingly, we fabricated the rGO device consisting of a sensor array capable of simultaneous detection of all three conformations of Aβ40, as described previously, and measured the ΔR_2_ values of the rGO sensors after addition of Aβ40 solutions (in 1% DMSO) with different incubation times (0 h, 4.5 h, 1 day, 5 days, and 10 days) (Figure 4a).

Figure 4a depicts the relative resistance changes of the rGO sensors after addition of the Aβ40 solutions. With no incubation, the ΔR_2_ value for monomeric Aβ40 was 5.2%, and those for oligomers and fibrils were below 1%, indicating that the majority of Aβ40 remained in the monomeric form. As the incubation time increased, the ΔR_2_ for monomeric Aβ40 rapidly decreased, and those for oligomers and fibrils increased gradually. For example, after incubation for 24 h, the ΔR_2_ values were 0.7% for monomers and 1.3% for both oligomers and fibrils. This could be attributed to the observation that the Aβ40 monomers must be consumed in order to form oligomers and fibrils. Thus, these data demonstrated that the portion of fibrillary Aβ40 continuously increased as the incubation time was in-creased. After 10 days of incubation, the ΔR_2_ values were 4.2% for oligomers and 4.3% for fibrils, that for monomeric Aβ40 was decreased to 0.1%. Thus, even after a large number of fibrils was formed, a significant number of oligomers still existed, although the monomers were severely depleted (Figure 4b). This trend was consistent with the results of TEM analysis (Figure 3a–c). From these data, we concluded that our rGO sensors may have great potential for monitoring the relative amounts of the three conformations of Aβ40 in solution.

For clinical application of our device, it is also of importance to verify whether each rGO sensor unit immobilized with a particular antibody specifically responds to its corresponding antigen (monomer, oligomer, or fibril) with-out any interference. Thus, we employed two model solutions to conduct selectivity tests. The first solution was the Aβ40 solution (A) with no incubation, which contained monomers in very large amounts and few or no oligomers and fibrils. The second solution was the Aβ40 solution (B) treated with microdialysis after 10 days of incubation. During microdialysis [27,28], most oligomers and monomers were eliminated by penetrating a semipermeable membrane, yielding a solution containing high amounts of fibrillary Aβ40. By mixing two model solutions with different ratios (A:B = 1:9, 5:5, and 9:1), three test solutions were fabricated. We measured the relative resistance changes of the rGO sensors when three test solutions added individually (Figure 4c). At A:B = 9:1, the ΔR_2_ for monomers was highest (1.9%) among the three conformations, and fibrillary Aβ40 showed the lowest signal (ΔR_2_ = 0.1%). At A:B = 1:9, however, the fibrillary Aβ40 showed the highest ΔR_2_ (3.8%), and both signals for monomers and oligomers were reduced, indicating that fibrils were the dominant conformation. As anticipated, all three conformations showed fairly discernable signals in the case of A:B = 5:5. These results suggested that each rGO sensor unit for a particular conformation had such excellent selectivity that there was nearly no interference by the other two conformations of Aβ40.

Next, we evaluated whether our device responded to proteins other than Aβ40. As a negative control, we used prostate-specific antigen (PSA). The results for this negative control are provided in Figure S4. All the rGO sensor units for the three conformations of Aβ40 exhibited no significant changes in ΔR_2_ (<0.5%) when the PSA solution (1 ng/mL) was added. From the above results, we concluded that our rGO device retained fairly good selectivity with respect to protein type and Aβ40 conformation.

### 3.4. Monitoring the Treatment Effects of Drugs on Disaggregation of Aβ40 Fibrils

**Figure 5:**
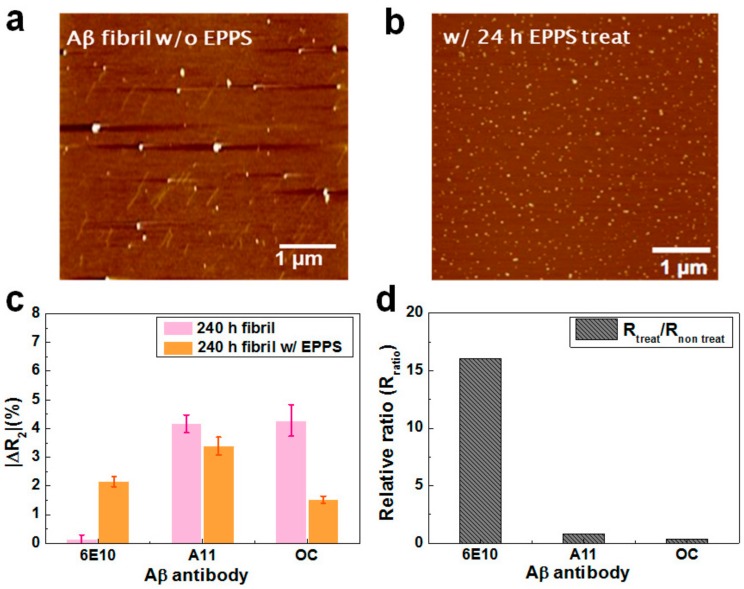
(**a**) Atomic force microscopy (AFM) images of Aβ40 samples incubated for 10 days without or (**b**) with 4-(2-hydroxyethyl)-1-piperazinepropanesulphonic acid (EPPS) treatment. The Aβ40 solution incubated for 10 days was treated with EPPS for 24 h and then analyzed by AFM. (**c**) The ΔR_2_ values of the sensors with respect to EPPS treatment time (no treatment and 24 h); (**d**) Relative ratios of ΔR_2_ values for monomers, oligomers, and fibrils.

Information regarding the effects of drugs on the integrity of Aβ40 aggregates is essential for evaluating the clinical applications of drugs in the treatment of AD. A main ad-vantage of our rGO device is the ability to monitor the extent of Aβ40 decomposition during drug treatment. In a recent study, Kim and coworkers reported that EPPS has the unique property of converting neurotoxic oligomers and fibrils into nontoxic monomers in vitro by directly binding to Aβ aggregates [18]. They showed that EPPS also had similar effects, even in an in vivo experiment using mice, exhibiting satisfactory improvement after treatment. In our experiment, Aβ40 samples with incubated for 10 days were treated with EPPS for 1 or 24 h. Aβ samples with or without EPPS treatment were then analyzed by atomic force microscopy (AFM). Topological images of Aβ samples with and without EPPS treatment are shown in Figure 5a,b. Large amounts of Aβ fibrils and oligomers appeared in the image of the sample with no EPPS, implying that the majority of Aβ existed in the fibrillary form. After treatment with EPPS for 24 h, however, a number of small particles (~10 nm in diameter) scattered, and there was a limited number of fibrils in the image. These data indicated that the fibrils and oligomers were effectively disaggregated into monomers after EPPS treatment for 24 h. 

To verify whether our device could precisely quantify the treatment effects of EPPS, we measured the ΔR_2_ values of rGO sensors with immobilization of three different antibodies after reaction of the aggregated and disaggregated Aβ40 sample. Before treatment of the 24 h sample, we confirmed the sensor reactivity with the fibril-enriched sample. We analyzed the Aβ40 solution after microdialysis using the rGO sensor (Figure S5). Based on this result, it was plausible that fibrils were the dominant species in the solution. Figure 5c depicts the relative resistance changes of the rGO sensors after addition of Aβ40 samples treated with or without EPPS (24 h). As we showed above, without EPPS, the majority of Aβ40 existed in the fibrillary conformation (ΔR_2_ = 4.3%), with few monomers (ΔR_2_ = 0.1%). After EPPS treatment for 24 h, the ΔR_2_ values of the rGO unit were 2.1% for monomers, 3.4% for oligomers, and 1.4% for fibrils, suggesting that the Aβ40 fibrils and oligomers rapidly disaggregated into monomers.

For a comparison, we calculated the relative ratio (R_ratio_) of the ΔR_2_ after EPPS treatment to the ΔR_2_ before treatment for each conformation, as shown in Figure 5d. The R_ratio_ for the monomers was markedly high (16.2), implying that after EPPS treatment, a very large amount of monomer was produced. In the other cases, however, the R_ratio_ was estimated to be 0.81 for oligomers and 0.35 for fibrils, indicating that considerable amounts of oligomers and fibrils were disaggregated into monomers. This trend was consistent with the results of AFM analysis and suggested that our rGO sensors were applicable as a drug screening system to precisely monitor the decomposition behavior of Aβ40 aggregates following the addition of particular chemicals. Moreover, our device may be applicable as a diagnostic system to estimate a patient’s condition through analysis of the patient’s blood or CSF.

## 4. Conclusions

In summary, we developed multi-functionalized rGO sensors that were composed of a multisensory array capable of simultaneous monitoring of three conformations of Aβ40. One rGO device included two reference sensor units and 10 individual sensor units for capturing the corresponding conformation of Aβ40. From the basic test of the performance of each unit, we found that each sensor exhibited a fairly good sensitivity and selectivity. Using our devices, the aggregation behavior of the Aβ40 with respect to the incubation was investigated through simultaneous monitoring of three conformations of Aβ40 in solution. From this analysis, we observed that fibrils and oligomers were actively formed as the incubation time was prolonged. In contrast, the Aβ40 solution with no incubation contained monomers in very large amounts, whereas both oligomer and fibrils were depleted. This trend was consistent with the results of TEM analysis. Moreover, we found that the decomposition characteristics of the Aβ40 fibrils could be measured after addition of a drug (EPPS), which caused disaggregation of the oligomers and the fibrils into monomers. This trend was also confirmed by the results of the AFM analysis conducted with the same samples.

Detailed information regarding the relative amounts of the three conformations of Aβ40 could provide us with vital clues to improve our understanding of the aggregation behaviors of Aβ40. This information may be useful in the diagnosis of AD or for predicting the prognosis of patients with AD. Changes in the relative amounts of the three conformations with time during drug screening may also provide essential information needed for the development of new drugs to cure AD. Notably, our rGO device was able to precisely monitor the relative amounts of the three con-formations of Aβ40 in solutions with different histories (e.g., incubation times and drug treatments). Accordingly, we believe that our rGO device has promising potential applications as a drug screening system to directly provide information following the addition of chemicals. Furthermore, the evident analytic results from present and previous studies have suggested that this sensor device can be utilized as an in vitro diagnostic tool to estimate conditions or progression of severe diseases via clinical samples that are oriented from patients’ body fluids, such as CSF, plasma, and urine. We are currently investigating these topics in our laboratory using our rGO sensor.

## Supplementary Materials

The following are available online at http://www.mdpi.com/1424-8220/18/6/1738/s1, Figure S1: Fabrication of the rGO sensors. Schematic illustrations of (a) the rGO coating by the MDD method and (b) the fabrication of rGO biosensors., Figure S2: Confirmations of the uniform immobilization of antibodies on the rGO surfaces. Resistance changes (ΔR_1_ values) were measured after the immobilization of (a) 6E10, (b) A11, and (c) OC., Figure S3. Thioflavin T (ThT) assay and AFM analysis of Aβ40 fibrils. (a) ThT fluorescent assay before and after incubation of the Aβ40 (10 ng/mL) solutions. (b) An AFM topological image of Aβ40 fibrils after incubation. Figure S4: Negative control with PSA protein., Figure S5: Effects of EPPS treatment on fibril-rich Aβ_40_ aggregates., Table S1: Comparison of Aβ sensing.
