# Supplementary material for: Multifunctionalized Reduced Graphene Oxide Biosensors for Simultaneous Monitoring of Structural Changes in Amyloid-β 40
Source: Sensors (Basel). 2018 May 28;18(6):1738. doi: 10.3390/s18061738 (PMC6022081; doi:10.3390/s18061738)
Supplement: Supplementary file 1 [file sensors-18-01738-s001.pdf]

sensors

Supplementary Information:

Multifunctionalized Reduced Graphene Oxide Biosensors for Simultaneous Monitoring of Structural Changes in Amyloid-β 40

Dahye Jeong, Jinsik Kim, Myung-Sic Chae, Wonseok Lee, Seung-Hoon Yang, YoungSoo Kim, Seung Min Kim, Jin San Lee, Jeong Hoon Lee, Jungkyu Choi, Dae Sung Yoon, Kyo Seon Hwang

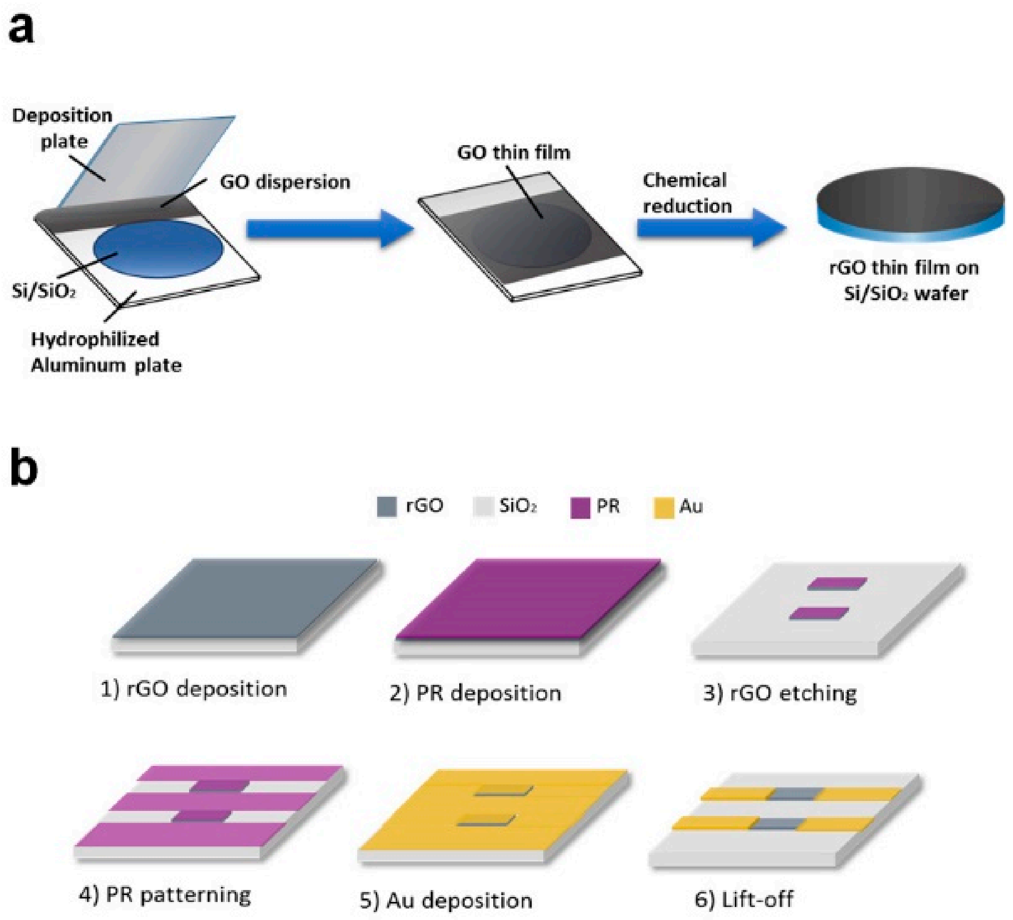

Figure S1. Fabrication of the rGO sensors. Schematic illustrations of (a) the rGO coating by the MDD method and (b) the fabrication of rGO biosensors.

A 4-inch Si/SiO_2 wafer was placed on an aluminum plate, and the deposition plate was positioned at an angle of 30°. Graphene oxide (GO) solution was added to the gap between the deposition plate and the wafer. The deposition plate was moved back and forth to form a uniform GO thin film on the wafer. The wafer was then dried on a hot plate at 100 °C. To obtain rGO, chemical reduction with HI acid vapor was performed, as shown in Supplementary Fig. 1a. The rGO deposition process is the basic step to fabricate the rGO biosensor. The photoresist was deposited on the rGO deposited SiO_2/Si wafer and developed using standard PR processes. rGO etching was conducted to form a pattern with the rGO sensor units with reactive ion etching in an O_2 gas

atmosphere. After the formation of rGO patterns, the PR was deposited again and developed to form gold (Au) electrodes that could be connected with rGO patterns. Subsequently, Au deposition was performed with an E-beam evaporator. The lift-off process was performed in acetone for 20 min to form Au electrodes.

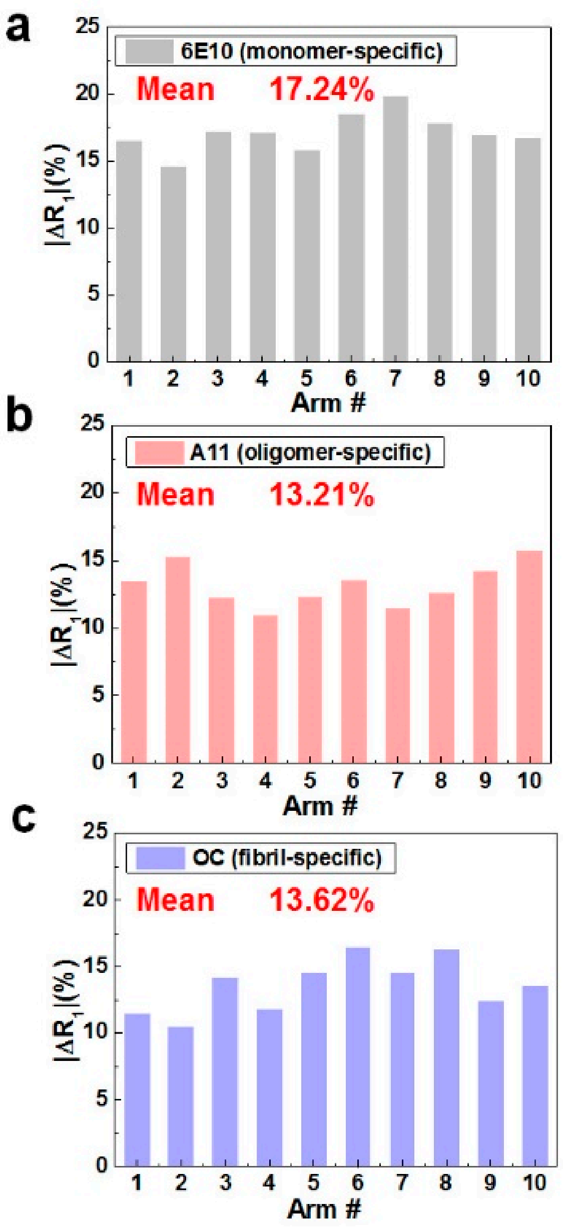

Figure S2. Confirmations of the uniform immobilization of antibodies on the rGO surfaces. Resistance changes (ΔR_1 values) were measured after the immobilization of (**a**) 6E10, (**b**) A11, and (**c**) OC.

After antibody immobilization, the ΔR_1 values of the rGO sensors, $(R_{ab} - R)/R$, were measured to verify whether the antibodies were uniformly immobilized on the rGO surfaces. Ten rGO sensor units of the devices were randomly selected for the uniformity test at each antibody immobilization. The CVs (coefficients of variance) were defined as σ (standard deviation)/A (average) × 100 and were within 10% for all three cases.

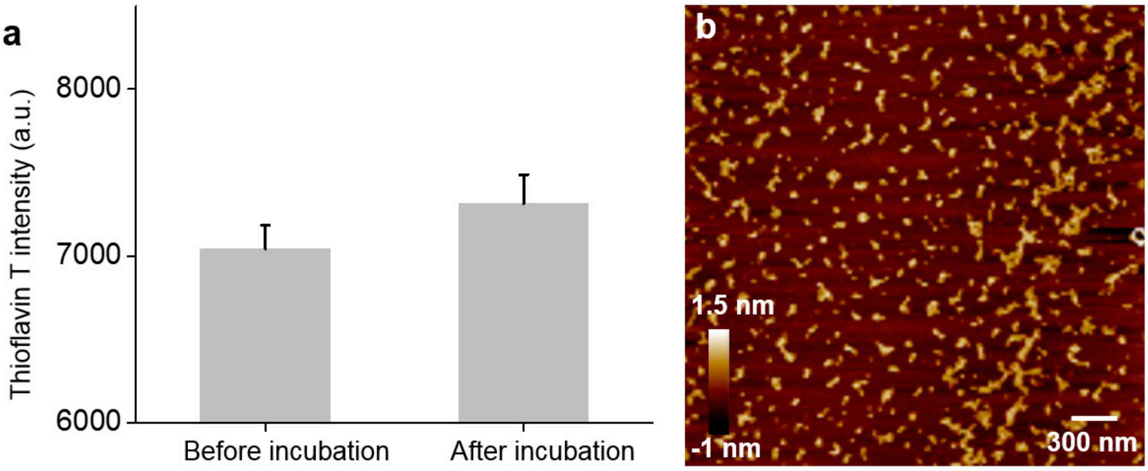

Figure S3. Thioflavin T (ThT) assay and AFM analysis of Aβ40 fibrils. (a) ThT fluorescent assay before and after incubation of the Aβ40 (10 ng/ml) solutions. (b) An AFM topological image of Aβ40 fibrils after incubation.

The ThT intensities of Aβ40 before and after incubation were 7,042 ± 142 and 7,313 ± 173 in a.u., respectively. The increased ThT intensity of the Aβ40 sample with incubation strongly supports the formation of β-sheets-rich aggregates (*i.e.*, fibrils) during incubation.

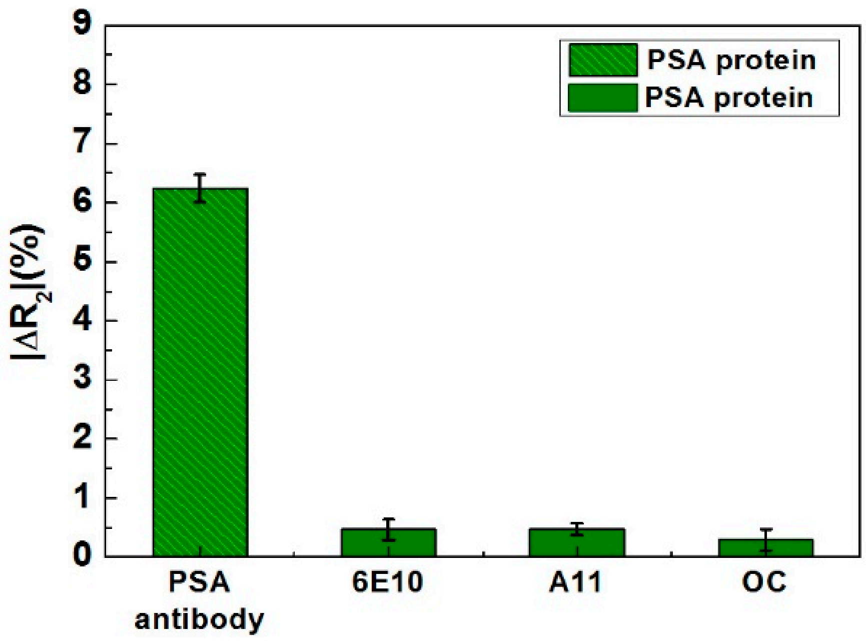

Figure S4. Negative control with PSA protein.

PSA protein (30C-CP1017; Fitzgerald), which exhibits non-specific binding to target antibodies, was utilized as a negative control. Antibodies (1 mg/mL) were immobilized on the rGO surfaces, and 1 ng/mL PSA protein was reacted with each antibody.

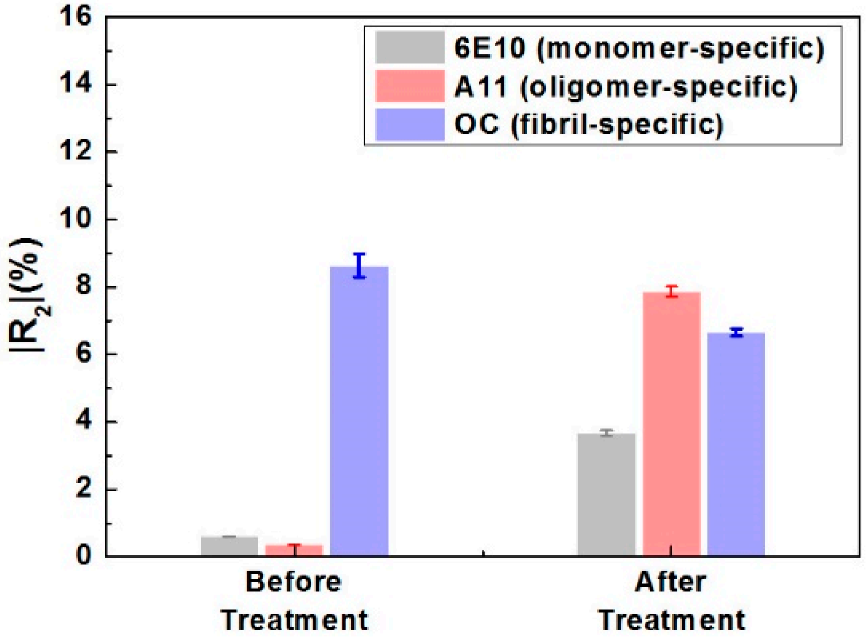

Figure S5. Effects of EPPS treatment on fibril-rich Aβ_{40} aggregates.

Microdialysis was conducted using Aβ_{40} solution after incubation for 10 days to prepare a fibril-rich Aβ_{40} solution. After the microdialysis, the Aβ_{40} solution was treated with EPPS for 24 h and then analyzed using the rGO sensors.

Table S1. Comparison of Aβ sensing.

Sensor Type	Aβ Types	Limit of Detection	Reference
rGO-FET	Aβ_{1-42}	1 fM	[1]
CNT-MESFET	Aβ_{1-42}	1 pg/mL	[2]
GO-based fluorescence	Aβ_{1-42} oligomers	10 nM	[3]
QCM	Aβ_{1-16}	50 pg/mL	[4]
DVP	A$\beta_{1-40/1-42}$	100 pM	[5]
Cyclic voltammetry	A$\beta_{1-16/1-40/1-42}$	20 pM	[6]
Cyclic voltammetry with N-doped graphene modified Au electrodes	Aβ_{1-42}	5 pg/mL	[7]
SERS	Aβ_{1-42}	500 fg/mL	[8]
SPR	Aβ_{1-42}	100 pg/mL	[9]
EIS	Aβ_{1-42} oligomer	1 pM	[10]
EIS with carbon disposable electrochemical printed chip	A$\beta_{1-40/1-42}$	10 pM	[11]
Square wave voltammetry	A$\beta_{1-40/1-42}$	20 nM	[12]
rGO based electrical sensor	Synthetic Aβand Aβ	100 fg/mL (synthetic Aβ)	[13]

	in neural exosomes		
**rGO based multi-pexing electrical sensor**	**$A\beta_{40}$ monomer, oligomer and fibril**	**100 fg/mL**	**In this work**

Abbreviations: rGO, reduced graphene oxide, CNT, carbon nanotube; FET, field effect transistor; MESFET, metal semiconductor field effect transistor; QCM, quartz crystal microbalance; DPV, Differential pulse voltammetry; SPR, surface plasmon resonance; SERS, surface enhanced Raman spectroscopy; EIS, electrochemical impedance spectroscopy; ELISA, enzyme-linked immunosorbent assay; CSF, cerebrospinal fluid.

References

1. Kurkina, T.; Sundaram, S.; Sundaram, R.S.; Re, F.; Masserini, M.; Kern, K.; Balasubramanian, K. Self-Assembled Electrical Biodetector Based on Reduced Graphene Oxide. *ACS Nano* **2012**, *6*, 5514–5520.
2. Oh, J.; Yoo, G.; Chang, Y.W.; Kim, H.J.; Jose, J.; Kim, E.; Pyun, J.-C.; Yoo, K.-H. A carbon nanotube metal semiconductor field effect transistor-based biosensor for detection of amyloid-beta in human serum. *Biosens. Bioelectron.* **2013**, *50*, 345–350.
3. Liu, L.; Xia, N.; Zhang, J.; Mao, W.; Wu, Y.; Ge, X. A graphene oxide-based fluorescent platform for selective detection of amyloid-β oligomers. *Anal. Methods* **2015**, *7*, 8727–8732.
4. Mustafa, M.K.; Nabok, A.; Parkinson, D.; Tothill, I.E.; Salam, F.; Tsargorodskaya, A. Detection of β-amyloid peptide (1–16) and amyloid precursor protein (APP_{770}) using spectroscopic ellipsometry and QCM techniques: A step forward towards Alzheimers disease diagnostics *Biosens. Bioelectron.* **2010**, *26*, 1332–1336.
5. Yu, Y.; Sun, X.; Tang, D.; Li, C.; Zhang, L.; Nie, D.; Yin, X.; Shi, G. Gelsolin bound β-amyloid peptides(1–40/1–42)_ Electrochemical evaluation of levels of soluble peptide associated with Alzheimer’s disease. *Biosens. Bioelectron.* **2015**, *68*, 115–121.
6. Liu, L.; Zhao, F.; Ma, F.; Zhang, L.; Yang, S.; Xia, N. Gelsolin bound β-amyloid $peptides_{(1–40/1–42)}$: Electrochemical evaluation of levels of soluble peptide associated with Alzheimer's disease. *Biosens. Bioelectron.* **2013**, *49*, 231–235.
7. Li, S.-S.; Lin, C.-W.; Wei, K.-C.; Huang, C.-Y.; Hsu, P.-H.; Liu, H.-L.; Lu, Y.-J.; Lin, S.-C.; Yang, H.-W.; Ma, C.-C.M. Non-invasive screening for early Alzheimer's disease diagnosis by a sensitively immunomagnetic biosensor. *Sci. Rep.* **2016**, *6*, 25155.
8. Demeritte, T.; Nellore, B.P. V.; Sinha, S.S.; Pramanik, A.; Ray, P.C. Hybrid Graphene Oxide Based Plasmonic-Magnetic Multifunctional Nanoplatform for Selective Separation and Label-Free Identification of Alzheimer's Disease Biomarkers. *ACS Appl. Mater. Interfaces* **2015**, *7*, 13693–13700.
9. Lee, Y.K.; Lee, K.-S.; Kim, W.M.; Sohn, Y.-S. Detection of Amyloid-β42 Using a Waveguide-Coupled Bimetallic Surface Plasmon Resonance Sensor Chip in the Intensity Measurement Mode. *PLoS ONE* **2014**, *9*, e98992-7.
10. Rushworth, J.V.; Ahmed, A.; Griffiths, H.H.; Pollock, N.M.; Hooper, N.M.; Millner, P.A. A label-free electrical impedimetric biosensor for the specific detection of Alzheimer’s amyloid-beta oligomers. *Biosens. Bioelectron.* **2014**, *56*, 83–90.
11. Lien, T.T.N.; Takamura, Y.; Tamiya, E.; Vestergaard, M.C. Modified screen printed electrode for development of a highly sensitive label-free impedimetric immunosensor to detect amyloid beta peptides. *Anal. Chim. Acta* **2015**, *892*, 69–76.
12. Prabhulkar, S.; Piatyszek, R.; Cirrito, J.R.; Wu, Z.-Z.; Li, C.-Z. Microbiosensor for Alzheimer’s disease diagnostics: detection of amyloid beta biomarkers. *J. Neurochem.* **2012**, *122*, 374–381.

13. Chae, M.-S.; Jeong, D.; Lee, S.M.; Heo, Y.; Kang, J.Y.; Lee, J.H. Enhancing surface functionality of reduced graphene oxide biosensors by oxygen plasma treatment for Alzheimer's disease diagnosis. *Biosens. Bioelectron.* **2017**, *92*, 610–617.
